# Lived Experiences and Technological Literacy of Heart Failure Patients and Clinicians at a Cardiac Care Centre in Uganda

**DOI:** 10.5334/aogh.2905

**Published:** 2020-07-28

**Authors:** Jason Hearn, Quynh Pham, Jeremy I. Schwartz, Isaac Ssinabulya, Ann R. Akiteng, Heather J. Ross, Joseph A. Cafazzo

**Affiliations:** 1 Institute of Biomaterials and Biomedical Engineering, University of Toronto, Toronto, ON, CA; 2 Centre for Global eHealth Innovation, Techna Institute, University Health Network, Toronto, ON, CA; 3 Institute of Health Policy, Management and Evaluation, Dalla Lana School of Public Health, University of Toronto, Toronto, ON, CA; 4 Section of General Internal Medicine, Yale University School of Medicine, New Haven, CT, US; 5 Uganda Initiative for Integrated Management of Non-Communicable Diseases, Kampala, UG; 6 Department of Medicine, Makerere University College of Health Sciences, Kampala, UG; 7 Uganda Heart Institute, Mulago Hospital, Kampala, UG; 8 Ted Rogers Centre for Heart Research, Peter Munk Cardiac Centre, University Health Network, Toronto, ON, CA; 9 Department of Medicine, University of Toronto, Toronto, ON, CA

## Abstract

**Background:**

Digital health could serve as a low-cost means of enabling better self-care in patients living with heart failure (HF) in resource-limited settings such as Uganda. However, digital health interventions previously deployed in such settings have been unsuccessful due to a lack of local patient and clinician engagement in the design process.

**Objective:**

To engage Ugandan HF patients and clinicians regarding their experiences with HF management and technology, so as to inform the future design of a digital health intervention for HF patients in Uganda.

**Methods:**

The study employed a convergent parallel mixed-methods design. Data collection was completed at the Uganda Heart Institute in Kampala, Uganda. Data were ascertained through a patient survey and semi-structured interviews completed with HF patients, caregivers, physicians, and nurses. A conventional content analysis approach was used to qualitatively examine interview transcripts.

**Findings:**

Survey data were collected from 101 HF patients (62 female/39 male, aged 54.2 ± 17.5 years). Nearly half (48%) disagreed that they knew what to do in response to changes in their HF symptoms. Almost all patients (98%) had access to a mobile device. Many patients (63%) identified as comfortable in using mobile money – a local set of services that use Unstructured Supplementary Service Data (USSD). Interviews were completed with 19 HF patients, three caregivers, seven physicians, and three nurses. Qualitative analysis revealed four clusters of themes: overdependence of patients on the clinic, inconvenience associated with attending the clinic, inconsistent patient self-care behaviours at home, and technological abilities that favoured USSD-based services.

**Conclusions:**

Ugandan HF patients possess unmet information needs that leave them ill-equipped to care for themselves. Future digital health interventions for this population should empower patients with HF-specific information and reassurance in their self-care abilities. Based on patient preferences, such systems should harness USSD technology with which most patients are already comfortable.

## Background

Cardiovascular disease (CVD) is the number-one cause of death globally [1], with over three-quarters of deaths associated with CVD occurring in low- and middle-income countries (LMICs) [2]. In Uganda, CVD accounts for 10% of all deaths, making it the most common noncommunicable cause of death in the country [3]. The burden of CVD in Uganda also appears to be increasing, with the number of deaths attributed to ischemic heart disease increasing by 13.4% between 2007 and 2017 [4].

The rising burden of CVD in LMICs has led to an increased prevalence of heart failure (HF) [5,6] – a common end result of CVD where the heart is unable to meet bodily demands for blood and oxygen [7]. Although there is currently no cure for HF, proper lifestyle changes, symptom management, and medical therapy can allow individuals diagnosed with this condition to enjoy an improved quality of life [8,9]. Accordingly, digital health interventions have recently been developed to simplify the process of managing and tracking the symptoms of HF [10,11,12,13,14,15,16,17,18,19]. One such intervention, which was developed alongside HF patients and clinicians living in Canada, has been shown to improve patient self-care and quality of life amongst Canadian HF patients [19,20].

Based on the success of digital health interventions in high-income countries, digital health presents a potential means of mitigating the growing burden of HF in LMICs. Due to the increasing degree of mobile phone penetration in LMICs [21], a digital health intervention could serve as a low-cost, scalable, and effective means of tracking, managing, and detecting the symptoms of patients living with HF. That said, various interventions previously deployed in LMICs have been unsuccessful due to a lack of local patient and clinician engagement in the design process [22]. With the goal of informing a future digital health intervention for use in Uganda, we engaged Ugandan HF patients and clinicians regarding their experiences with HF management and mobile phone technology.

## Methods

This study was conducted at the Uganda Heart Institute (UHI) – a cardiac care centre located in Kampala, Uganda. The study employed a convergent parallel mixed-methods design [23]. Qualitative and quantitative data were collected concurrently through semi-structured interviews and patient surveys, respectively. The emergent datasets were analyzed individually using a conventional content analysis for the qualitative data [24] and a statistical analysis for the quantitative data. A merged analysis was then used to compare content areas represented in each dataset, allowing for conclusions to be drawn regarding patient and clinician experiences with HF management and mobile phone technology.

### Ethics Approval and Consent to Participate

Ethics approval was obtained from the Uganda National Council of Science and Technology (HS 2364), the Makerere University School of Medicine Research and Ethics Committee (REF 2017–076), and the University Health Network Research Ethics Board (ID# 14-7510). All study participants were given a standardized explanation of the study protocol, including potential risks and benefits, and subsequently consented to participate by either signing or providing a fingerprint on a consent form.

### Study Population

Patients and clinicians were recruited from UHI. Patient participants were considered eligible if they had previously been diagnosed with HF by a physician at UHI. A second eligibility criterion was that the patient spoke either English or Luganda, or that they had an accompanying caregiver that could interpret dialogue from a third language into English. Patients unable to participate due to the severity of their illness were asked whether their caregiver could speak on their behalf. Clinician participants were considered eligible if they were a physician or nurse with direct experience caring for HF patients at UHI.

### Data Collection

A survey was deployed amongst HF patients at UHI. The survey contained three sections: *Patient Information, Patient Opinion*, and *Medical Information*. The first two sections were completed by patients, whereas the final section was filled in by a consulting physician. The patient-filled portion of the survey included demographic information, self-reported health status, HF self-care behaviours, access to technology, and comfort with different communication channels. For the opinion-based items, which were loosely based on a previous questionnaire deployed in Canada [25], patients were asked to rate their level of agreement on a five-point Likert scale. The elicited medical information included time since HF diagnosis, number of annual visits to a HF clinic, New York Heart Association class, left ventricular ejection fraction, primary cause of HF, and comorbidities.

Semi-structured interviews were completed with a separate set of HF patients, caregivers, physicians, and nurses at UHI. Participants were asked about their experiences with the healthcare system, HF management, and technology. The line of questioning was guided by a series of open-ended prompts (see **Supplementary Materials 1 and 2**), allowing for focused yet natural discussions regarding the three main topics. A research assistant was present for each patient interview and offered interpretation services for patients that felt more comfortable speaking in Luganda. For participants that did not speak either English or Luganda, interviews were completed with the patient’s caregiver providing English interpretations of the patient’s dialogue. Clinician interviews were conducted in English. The interviews typically lasted between 15 and 45 minutes, and each interview was recorded for post hoc analysis.

### Quantitative Analysis

Survey data were inputted into Microsoft Excel v16.36 (Microsoft, Redmond). Categorical data were summarized using count and percentage. Numerical data were represented using mean and standard deviation. For opinion-based survey items, responses provided using the five-point Likert scale were summarized using mean and standard deviation. The percentages of participants that were in agreement (i.e. responded *Agree* or *Strongly Agree*), neutral (i.e. responded *Neither Agree or Disagree*), and in disagreement (i.e. responded *Disagree* or *Strongly Disagree*) were also reported for each survey item. The significance of differences in agreement between two related survey items (e.g. comfortability with two different methods of communication) was assessed by comparing the percentage of participants that were in agreement with each item using McNemar’s test for paired nominal data [26].

### Qualitative Analysis

Audio recordings of each interview were professionally transcribed for analysis. A conventional content analysis approach was used to examine the transcripts [24]. Two members of the research team (JH and QP) independently read, interpreted, and coded the transcripts in sets of five or six. After each set, the two researchers would meet to compare results and to discuss common themes before proceeding to the next group of transcripts. This process was repeated until all transcripts had been coded and a consensus had been reached on the main themes emerging from the analysis. All qualitative analyses were completed using NVivo v12.0.0 (QSR International, Melbourne).

## Results

### Patient Survey

A total of 101 patients (62 female/39 male, aged 54.2 ± 17.5 years) completed a survey. Table 1 summarizes the demographic and clinical characteristics of the surveyed patients. Amongst the participants, 62% reported having no monthly household income, and only 8% reported a monthly income greater than 500,000 Ugandan shillings. Over 60% of individuals possessed a primary school education or less, while 30% of patients self-identified as illiterate. More than half of patients (54%) were New York Heart Association class III or IV. The primary HF causes were cardiomyopathy (42%), hypertension (24%), and rheumatic heart disease (17%). Table 2 describes technology access amongst the surveyed population. A mobile device was accessible (either owned or borrowed) by 98% of individuals, while only 16% had access to a smartphone. Only 12% and 17% of individuals had access to a weighing scale and blood pressure cuff, respectively.

**Table 1:** Demographic and clinical characteristics of surveyed patients, where SD = standard deviation.

Variable		Count/*Mean*	Percentage/*SD*

Sample size		101	
Age (years)		*54.2*	*17.5*
Sex	Male	39	38.6%
	Female	62	61.4%
Ethnicity	Ganda	44	43.6%
	Munyankole	10	9.9%
	Musoga	10	9.9%
	Other	37	36.6%
Marital status	Single	16	15.8%
	Married	52	51.5%
	Separated/Divorced	10	9.9%
	Widowed	23	22.8%
Number of people in home		*5.5*	*3.3*
Education achieved	No study	11	10.9%
	Primary	51	50.5%
	Secondary	20	19.8%
	University	19	18.8%
Literacy level	Illiterate	30	29.7%
	Some reading/writing	22	21.8%
	Fully literate	49	48.5%
Monthly household income	No income	63	62.4%
	<50,000 USh	8	7.9%
	50,000–500,000 USh	22	21.8%
	500,000–1,000,000 USh	7	6.9%
	>1,000,000 USh	1	1.0%
Employment	Employed	11	10.9%
	Self-employed	28	27.7%
	Unemployed	57	56.4%
	Retired	5	5.0%
Self-reported health status	Poor	30	29.7%
	Fair	58	57.4%
	Good	13	12.9%
	Excellent	0	0.0%
Years with heart failure		*2.5*	*3.0*
New York Heart Association class	I	6	5.9%
	I/II	2	2.0%
	II	34	33.7%
	II/III	2	2.0%
	III	44	43.6%
	IV	11	10.9%
	N/A	2	2.0%
Left ventricular ejection fraction		*39.8%*	*15.7%*
Primary cause of heart failure	Cardiomyopathy	42	41.6%
	Hypertension	24	23.8%
	Rheumatic heart disease	17	16.8%
	Other	16	15.8%
	N/A	2	2.0%

**Table 2:** Patient responses to technology-related survey items.

Variable		Count	Percentage

Sample size		101	
Access to weight scale	Yes (at home)	4	4.0%
	Yes (from a friend)	4	4.0%
	Yes (from work)	3	3.0%
	Yes (from street)	1	1.0%
	No	89	88.1%
Access to blood pressure cuff	Yes (at home)	8	7.9%
	Yes (from a friend)	6	5.9%
	Yes (from work)	3	3.0%
	No	84	83.2%
Access to mobile phone	Yes (own phone)	90	89.1%
	Yes (borrow from a friend)	9	8.9%
	No	2	2.0%
Type of mobile phone	Smartphone	16	15.8%
	Non-smartphone	84	83.2%
	No phone	2	2.0%

Responses to opinion-based survey items (Table 3) revealed that 54% of patients agreed that they need to weigh themselves every day, while 77% agreed that it is important to check their blood pressure at home. Approximately half (52%) of respondents agreed that they were confident in their ability to care for themselves. Nearly half (48%) disagreed that they knew what to do in response to changes in their HF symptoms, with 47% of patients responding with *Strongly Disagree*. A significantly larger proportion of patients agreed that they were comfortable using their phone for mobile money services (63%), when compared to using their device for short message service (SMS) messaging (46%, *P* < 0.001) and interactive voice response systems (47%, *P* = 0.002).

**Table 3:** Patient responses to opinion-based survey items, where SD = standard deviation.

Survey Item	Mean ± SD	% Agree*	% Neutral^†^	% Disagree^‡^

I need to weigh myself every day at home.	3.2 ± 1.4	54.5%	14.9%	30.7%
It is important to check my blood pressure at home.	3.8 ± 1.3	77.2%	5.9%	16.8%
I am confident in my ability to care for myself with regard to my heart failure condition.	3.4 ± 1.2	52.5%	28.7%	18.8%
I know what to do in response to changes in my heart failure symptoms (e.g. shortness of breath or fluid buildup in legs).	2.8 ± 1.7	51.5%	1.0%	47.5%
I feel comfortable using a mobile phone to send and receive SMS messages.	2.7 ± 1.8	45.5%	2.0%	52.5%
I feel comfortable using a mobile phone to interact with an interactive voice response system.	2.7 ± 1.8	46.5%	1.0%	52.5%
I feel comfortable using a mobile phone for the purposes of mobile money.	3.2 ± 1.7	63.4%	1.0%	35.6%

* % Agree = percentage of responses as either Agree (4) or Strongly Agree (5). ^†^ % Neutral = percentage of responses as Neither Agree or Disagree (3). ^‡^ % Disagree = percentage of responses as either Disagree (2) or Strongly Disagree (1).

### Patient & Clinician Interviews

#### Overview of Interview Results

**Figure 1:**
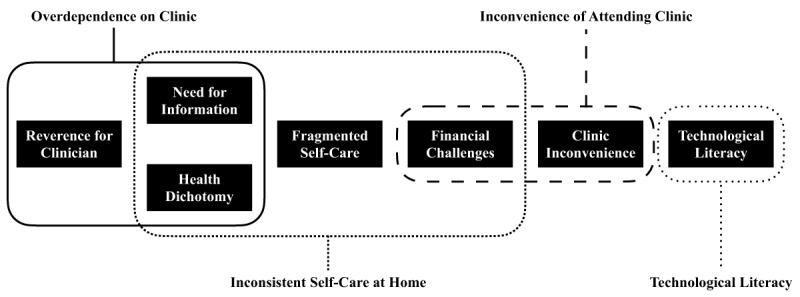
Summary of the seven themes and four theme clusters emerging in the qualitative analysis.

Semi-structured interviews were completed with 19 HF patients, three informal caregivers, seven physicians, and three nurses. Seven themes emerged in the qualitative analysis of the patient and clinician interviews. Correlated themes were grouped to form a hierarchical theme structure involving four theme clusters: *Overdependence on Clinic, Inconvenience of Attending Clinic, Inconsistent Self-Care at Home*, and *Technological Literacy* (Figure 1). It is worth noting that three themes were categorized within two separate theme clusters, given the multifactorial contributions of these themes to patients’ lived experiences.

#### Theme Cluster 1: Overdependence on Clinic

The first theme cluster was an apparent overdependence of patients on the clinic. Patients demonstrated a dependence on the clinic for virtually all information related to their condition and its management. In many cases, the clinic appeared to be the lone source of HF-specific information available to the patient.

*[The patient] needs more healthy teachings. Because he doesn’t know much about what to do. He has not much information on how to take care of himself. So, they need more… healthy teachings*.

Patient 10 (via interpreter)

This overdependence on the clinic was also evidenced by the fact that very few patients were informed on how to alter self-care behaviours in response to symptoms. As an example, only three of the interviewed patients demonstrated knowledge of a common HF self-care behaviour: taking extra diuretics in response to fluid overload. Instead, patients often reported that they would simply come to the clinic if they noticed any change in their symptoms.

*[The patient and I] cannot change the medication by ourselves. We have to go to the doctor and then he’ll tell you what’s happening and then the doctor himself decides to change the medication. Because, as a layperson, I cannot decide to change my medication. That’s why… when I explained that [the patient] was coughing, having difficulty breathing, she had to rush to see the doctor*.

Caregiver 2

In addition to the dependence on the clinic for information, patients were also found to be heavily reliant on clinicians to provide comfort and peace of mind regarding their condition. Patients often described coming to the clinic with fears and anxieties associated with their diagnosis. In particular, it was noted that patients frequently viewed and described their health using dichotomous language. Patients would often describe themselves as either healthy or dying, with little acknowledgement of the state of health in between.

*Sometimes, [the patient] feels better. But when [a symptom] comes up, she feels, “ah, the life is gone”*.

Patient 3 (via interpreter)

*The moment I was told about the sickness, it was like, tomorrow I’m going to die*.

Patient 1

As expected, patients would typically report to the clinic when their self-perceived health status tended towards the negative half of this health dichotomy. However, when given the opportunity to discuss their symptoms with a physician, patients would often leave the clinic with confidence that their condition was, in fact, manageable.

*After talking to the doctor, that’s when [the patient] feels that she will be well… Before, she’s scared. She thinks that she might die. But when she reaches the clinic, she feels that she will be well*.

Patient 14 (via interpreter)

The theme of an apparent expectancy on physicians to provide hope also emerged in the clinician interviews. Clinicians noted that the relationship between the physician and the patient remains largely one-sided in Uganda. This paternalistic relationship allows clinicians to play a large role in influencing changes in health behaviours; however, it also places an immense burden on clinicians to be the source of both information and comfort in their patients’ lives.

*I think the patients look at you like you’re going to provide hope. You provide hope, yes. But they expect you to provide a cure, especially if it’s young patients. … The patients look at you that you’re going to solve their problem and sometimes we’re not able*.

Physician 2

#### Theme Cluster 2: Inconvenience of Attending Clinic

The second cluster of themes highlighted the significant degree of inconvenience associated with patient visits to the clinic. Patients frequently described the immense financial burden associated with a typical clinic visit. This burden included direct treatment costs (e.g. consultations, investigations, medications), as well as indirect expenses associated with the often-long distance travelled to attend the clinic (e.g. transportation, food, accommodations). These financial costs were typically incurred either directly by patients or by family members providing financial support.

Another concern voiced by patients was the large time cost required to attend the clinic. Several patients reported arriving at the clinic in the early morning and not departing until the late afternoon. Some patients had previously waited in the clinic for an entire day, only to be told to return the following day to be seen by a clinician. This time cost was even larger for patients living outside of Kampala, who also had to factor in time for travel both to and from the clinic. For many patients, this inconvenience also led to time lost from work, further adding to the financial challenges listed above.

*We come early… You’d imagine that if we came early, we would be served early. But sometimes, that’s not the case. You come very early, we leave our places and we’re here by 6:00am, 5:30am. … People will leave at times disgruntled… Like today, we found some people who were not worked on yesterday, and they had to be worked on today*.

Patient 17

These frustrations with long waiting periods were shared by clinicians, many of whom insisted that their greatest challenge was the sheer number of patients coming to the clinic. Due to the large volume of patients being seen each day, several clinicians admitted that they were often unable to provide their patients with a complete picture of the HF condition.

*We are overwhelmed. So many patients, and you find yourself… you cannot even manage to have a 15-minute talk with a patient. Sometimes, I would say that we… we don’t give all… what the patients would need from us, because of all these issues we are dealing with*.

Nurse 1

A further frustration held by clinicians was the fact that many patients report to the clinic far too late. Clinicians claimed that patients often delay visiting the clinic until they present with an adverse event, at which point the heart is at risk of experiencing irreparable damage. Many clinicians attributed these late presentations to the financial and time costs associated with treatment, while others believed the delays to be caused by limited patient understanding of the condition and its severity.

*Our patients take long to come back to the hospital. When they go into failure, they try this, they try this, they try that, and by the time they come back, they are very very sick*.

Physician 1

#### Theme Cluster 3: Inconsistent Self-Care at Home

The third theme cluster centred around the lack of continuity in patient self-care behaviours. Instead of self-care being continuous with minimal disruptions, it was often described by patients as being cyclical in nature. As described previously, patients typically come to the clinic in response to changes in their symptoms or an adverse event, and subsequently leave the clinic with updated medications and newfound confidence in their self-care behaviours. Following a clinic visit, patients generally experience a substantial improvement in their state of health. However, patients often reported that their effective self-care behaviours then deteriorate over time. The most commonly cited cause of this breakdown in self-care was patients finishing a medication and not refilling it immediately, often due to a lack of funds or an incomplete understanding of the drug’s utility. This lapse in self-care is then generally followed by a regression in patients’ health. Naturally, worsening patients return to the clinic, restarting the cycle and further reinforcing the aforementioned burden on the clinic.

*When [the patient] goes [to the clinic]… she goes there when the whole body is swelling. When she goes there, they give her treatment, they give her medication. She comes home, takes the drugs and then in the process of taking the drugs, I think she does too much urinating and then the body reduces. So, she continues taking, taking until the drugs are finished and when they are finished, then she again worsens, then she goes back… When she’s not doing well, she goes to that clinic, they give her treatment, she takes that treatment. Then, when she feels better, she leaves the treatment*.

Patient 5 (via interpreter)

Patients reported several financial challenges associated with adequately caring for themselves at home. Many patients reported that, even upon leaving the clinic, they could not afford all of the medications prescribed by their clinicians. Patients noted that diuretics were often the only medications available to them at no cost, with most medications having to be purchased from the clinic or an external pharmacy. The financial challenges also expanded into other aspects of the HF self-care regimen. Many patients reported that they were unable to adhere to dietary recommendations received at the clinic, given the increased cost and decreased availability of the suggested foods.

*I was advised to take a lot of greens by the doctors here. At times, it becomes difficult also to get these greens around in markets. You may get them there, but then you may not get the money*.

Patient 12

In addition to the financial challenges faced by patients, clinicians also attributed the fragmented self-care to patients’ limited understanding of the severity of the HF condition. Specifically, clinicians noted that many patients fail to acknowledge the severity of their condition and hold firm to the belief that they will eventually be cured of HF. This expectation of a cure for HF emerged in several patient interviews.

Is it possible that heart failure can go, and I get healed?

Patient 4

After taking these drugs, do you think there will be a time when I’ll be completely… not again using these drugs?

Patient 2

If I don’t eat the beef, as the doctor says, if I don’t eat the pork, as the doctor says, and I don’t eat all that fatty stuff… Does [heart failure] remain in my life?

Patient 18

Further gaps in understanding of the HF condition and its management were highlighted in the patient interviews. Most patients did not seem to fully understand the causes of their various HF symptoms. Very few patients mentioned exercise as being a part of their self-care regimen. While some patients noted dietary recommendations from their clinicians, such as avoiding foods high in salt and fat, many patients emphasized medication adherence as the sole method of managing their condition at home.

*So, [the patient] hasn’t taken much care about herself so far. The way she has been managing her heart failure is to take drugs only*.

Patient 14 (via interpreter)

#### Theme Cluster 4: Technological Literacy

The final cluster of themes centred on the technological literacy of the interviewed patients. While virtually every patient had access to a mobile phone, most patients possessed a low-cost non-smartphone. It became immediately clear that patients’ preferred method of communication was calling. Very few patients reported using SMS messaging, and virtually all patients reported a preference for calling over SMS.

*So, [the patient] knows how to call, to make calls. But texting, no*.

Patient 16 (via interpreter)

*I basically call. … Because I’ll text you and it’s in your pocket, or in your bag, and if I sent it at midnight, they miss it. So, I’d rather call and disturb people*.

Patient 1

Internet and social media usage were found to be quite rare amongst the patient population. However, almost all patients had experience using a mobile money service on their device. Most patients reported using mobile money independently, while the remainder had at least used the system with support from a friend, a family member, or a mobile money service provider. Furthermore, patients frequently reported that they found mobile money easy to use on their mobile devices. Even patients who disliked or were uncomfortable with SMS messaging appeared to use mobile money with ease.

*[The patient] uses her phone mostly for calling and receiving. She doesn’t know how to text and send messages. And also, she knows how to use her mobile money. If someone sends, she knows how to operate it*.

Patient 8 (via interpreter)

Interestingly, low socioeconomic status appeared to be not so much a barrier to mobile money usage, but a factor that increased the likelihood of an individual having experience with the system. This unexpected result was caused by the fact that indigent, rural-dwelling individuals would often have their family send them funds via mobile money to cover the high costs of medication.

*Normally, if someone is sending me assistance, I don’t have to [use a motorcycle] or taxi. I can say, put it on my phone*.

Patient 1

*I have my sons who are within [Kampala]… So, I used to get money from them through my mobile money*.

Patient 12

## Discussion

The qualitative themes that emerged in this analysis offer important insight regarding the intricacies of HF management at a cardiac care centre in Kampala, Uganda. Firstly, it is important to acknowledge the manner in which the emergent theme clusters affect one another, as well as the challenges that result from these interactions. The large patient dependence on the clinic for HF-specific information and emotional support (Theme Cluster 1) leads to challenges for both patients and clinicians. Because the clinic is tasked with an immense patient volume (Theme Cluster 2), clinicians are forced to decide between a) providing a complete picture of HF management at the cost of seeing fewer patients or b) efficiently highlighting the critical aspects of HF management to an increased number of patients. As the clinicians – through no fault of their own – generally opt for the latter of the two options, patients end up with an incomplete understanding of their condition and its necessary self-care behaviours (Theme Cluster 3). Unfortunately, these unmet information needs presumably result in increased dependence (Theme Cluster 1) and patient burden (Theme Cluster 2) on the clinic, which in turn creates a vicious cycle.

One key finding in the analysis was a failure to empower HF patients to adequately care for themselves. This theme emerged in the interview phase and was supported by the large proportion of surveyed participants who disagreed that they know what to do in response to changes in their symptoms. Though patients should feel free to visit the clinic when they experience abrupt changes in their HF symptoms, study participants did not appear to have the knowledge required to care for themselves even if they had the desire to do so. This finding correlated with results from a 2017 study by Namukwaya et al., who found that “one of the greatest unmet needs from [Ugandan HF] patients was for information on the illness, its treatment, prognosis and how to self-care” [27].

The unmet information needs and limited self-care abilities amongst the Ugandan HF patients present a clear target for a future digital health intervention. Specifically, it appears that the target population could benefit from a system that provides patients with HF-specific information and reassurance in their self-care abilities. By empowering patients with information regarding their condition and its management, such a system could lead to several potential benefits, namely increased self-care efficacy, improved quality of life, fewer clinic visits, and reduced information requirements during patient-clinician interactions.

Based on the technological literacy reported by patients, the ideal platform for such an intervention would resemble existing mobile money services in Uganda. These services use Unstructured Supplementary Service Data (USSD), a communication protocol that is accessible from any GSM-enabled mobile device (i.e. smartphone or non-smartphone). USSD creates an open connection between the user’s device and a network or server, allowing the user to navigate through a decision tree by inputting numbers that correspond to desired actions [28]. The workflow of a USSD-based system resembles interactive voice response systems commonly used in North America; however, the lists of options are presented to the user in the form of text rather than being spoken aloud by an automated system. In the presented study, USSD-based mobile money services appeared to be well-adopted amongst participants of all socioeconomic classes. This finding reinforced Wyche and Murphy’s claim that USSD-based systems allow for the reaching of individuals at the “bottom of the pyramid” [29]. Their claim appears to hold true in the presented study population, not only because of the low technological requirements of USSD referenced by the authors [29], but also because of the apparent prevalence of mobile money usage amongst those financially dependent on friends and family. This existing familiarity with USSD technology and its functionality could greatly mitigate the usability challenges associated with designing a system specifically for users of low socioeconomic status. The compatibility of USSD with both smartphones and non-smartphones also promotes digital inclusion, and could limit accessibility issues often associated with new healthcare technologies [30]. As a result of these potential benefits, a small number of digital health interventions currently deployed in LMICs are using USSD [31,32,33], including a recent USSD-based intervention developed to track COVID-19 symptoms in Ghana [34]. As the identified interventions primarily focus on infectious disease tracking and maternal-fetal medicine, there is a need for further research into the applicability of USSD to digital health interventions for chronic disease management.

In addition to the various findings of the research study, it is important to acknowledge the numerous benefits that resulted from early engagement of local patients and clinicians. While a user-centred design process has been shown to be imperative for the success of digital health interventions in high-income countries [19], it remains underutilized in global health initiatives [22]. By engaging the target population, the authors gained an appreciation of the various challenges faced by HF patients and clinicians in Uganda, as well as the technological channels available to the eventual users of the proposed digital health intervention. The discovery of the patient-preferred USSD platform effectively demonstrates the benefit of early and direct end-user engagement.

The study is not without limitations. All presented information was collected at a semi-private cardiac care centre, which may have skewed patient-related data towards HF patients with a means of paying for cardiac services. This study population also limited the analysis to individuals that had made the decision to seek treatment at a cardiac clinic. Accordingly, the views of the study population may not be representative of the Ugandan HF community as a whole. The qualitative analysis was also limited by the fact that neither of the two reviewers were native Ugandans, which may have led to a lack of context in reviewing the transcripts. This final limitation was mitigated through consistent feedback, consultation and oversight from the two Ugandan investigators included in this research study.

## Conclusions

In Uganda, HF presents a large and increasing burden on both patients and the healthcare system. As digital health interventions have been shown to improve self-care efficacy and quality of life amongst HF patients living in high-income countries, such interventions could play a role in mitigating the burden of HF in Uganda. We interviewed and surveyed Ugandan HF patients and clinicians regarding HF management and technological literacy. Qualitative analysis identified an overdependence of patients on the clinic, an inconvenience associated with clinic visits, inconsistent patient self-care behaviours at home, and technological abilities that favoured USSD-based services. The patient survey supported themes emergent in the interviews, specifically a self-perceived inability for patients to adequately care for themselves, as well as a preference for the USSD platform. Based on the results, future digital health interventions for HF patients in Uganda should incorporate USSD technology and focus on empowering patients to better care for themselves.

## Data Accessibility Statement

The data collected and analyzed in the presented study are available from the corresponding author on reasonable request.

## Additional File

The additional file for this article can be found as follows:

10.5334/aogh.2905.s1 Supplementary Material 1. Interview guide used in patient interviews.

10.5334/aogh.2905.s2 Supplementary Material 2. Interview guide used in clinician interviews.

## Abbreviations

**CVD** Cardiovascular disease

**HF** Heart failure

**LMICs** Low- and middle-income countries

**SMS** Short message service

**UHI** Uganda Heart Institute

**USSD** Unstructured Supplementary Service Data
