# Supplementary material for: Lived Experiences and Technological Literacy of Heart Failure Patients and Clinicians at a Cardiac Care Centre in Uganda
Source: Ann Glob Health. 2020 Jul 28;86(1):85. doi: 10.5334/aogh.2905 (PMC7413178; doi:10.5334/aogh.2905)
Supplement: Supplementary Material 1. — Interview guide used in patient interviews. [file agh-86-1-2905-s1.pdf]

1 Supplementary Materials

Supplementary Material 1: Interview guide used in patient interviews

Introduction (~3 minutes)

1. Personal introduction and explanation of research purpose.
2. Explanation of how collected information will be protected and used.
3. Answering of any questions from participant.

Interview (~30 minutes)

Healthcare (~10 minutes)

1. "Tell me about your experience here at the clinic."
2. "How would you describe your relationship with your heart failure doctors and nurses?"
    a. PROMPT: "How could this relationship be improved?"

Heart failure management (~15 minutes)

1. "How do you currently manage your heart failure condition?"
    a. PROMPT: "How do you change your management in response to symptoms (e.g. shortness of breath or fluid buildup in legs)?"
2. "How confident are you in taking care of yourself with respect to your heart failure condition?"
    a. PROMPT: "What causes you anxiety with respect to your condition?"
    b. PROMPT: "What aspect of self-care do you find the hardest?"
3. "What do you think would help you take better care of yourself with respect to your heart failure condition?"

Technology (~5 minutes)

1. "How do you currently use your mobile phone?"
    a. PROMPT: "What communication channels do you use most frequently (e.g. text messaging, calling, internet or mobile money)?"
    b. PROMPT: "How comfortable do you feel in using your mobile device?"

Conclusion (~3 minutes)

1. "Is there anything else you would like to mention with respect to healthcare, heart failure management or technology that we didn't get a chance to discuss?"
2. Expression of gratitude for participation.
3. Explanation of next steps in the research project.

Target total time range: 30–45 minutes
