# Supplementary material for: Lived Experiences and Technological Literacy of Heart Failure Patients and Clinicians at a Cardiac Care Centre in Uganda
Source: Ann Glob Health. 2020 Jul 28;86(1):85. doi: 10.5334/aogh.2905 (PMC7413178; doi:10.5334/aogh.2905)
Supplement: Supplementary Material 2. — Interview guide used in clinician interviews. [file agh-86-1-2905-s2.pdf]

Supplementary Material 2: Interview guide used in clinician interviews

Introduction (~3 minutes)

1. Personal introduction and explanation of research purpose.
2. Explanation of how collected information will be protected and used.
3. Answering of any questions from participant.

Semi-structured portion (~16 minutes)

Healthcare (~4 minutes)

1. "How would you describe your relationship with your heart failure patients?"
    a. PROMPT: "How could this relationship be improved?"

Heart failure management (~7 minutes)

1. "How do you feel about your patients' ability to manage their heart failure condition?"
    a. PROMPT: "What aspects of care do your patients struggle with the most?"
2. "What do you think would allow your patients to more effectively self-manage their heart failure condition?"

Technology (~5 minutes)

1. "How would you feel about a mobile phone-based system for remote patient monitoring?"
    a. "What benefits would you expect?"
    b. "What challenges would you expect?"

Conclusion (~3 minutes)

1. "Is there anything else you would like to mention with respect to healthcare, heart failure management or technology that we didn't get a chance to discuss?"
2. Expression of gratitude for participation.
3. Explanation of next steps in the research project.

Target total time range: 15–30 minutes
